# Estimating disease burden using national linked electronic health records: a study using an English population-based cohort.

**DOI:** 10.12688/wellcomeopenres.19470.2

**Published:** 2024-06-19

**Authors:** Robert W. Aldridge, Hannah E. R. Evans, Alexei Yavlinsky, Alireza Moayyeri, Krishnan Bhaskaran, Rohini Mathur, Kelvin P. Jordan, Peter Croft, Spiros Denaxas, Anoop D. Shah, Ruth M. Blackburn, Henrik Moller, Edmond S. W. Ng, Andrew Hughes, Sebastian Fox, Julian Flowers, Jurgen Schmidt, Andrew Hayward, Ruth Gilbert, Liam Smeeth, Harry Hemingway

**Affiliations:** 1 Institute of Health Informatics, University College London, London, England, NW1 2DA, UK; 2 Faculty of Epidemiology & Population Health, London School of Hygiene and Tropical Medicine, London, WC1E 7HT, UK; 3 Wolfson Institute of Population Health, Queen Mary University of London, London, England, EC1M 6BQ, UK; 4 School of Medicine, Keele University, Staffordshire, England, ST5 5BG, UK; 5 Cancer Epidemiology & Population Health, King's College London, London, England, WC2R 2LS, UK; 6 Department of Health and Social Care, Office for Health Improvement and Disparities, London, SW1H 0EU, UK; 7 Institute of Epidemiology and Health Care, University College London, London, England, WC1E 6BT, UK

**Keywords:** burden of disease, electronic health records

## Abstract

**Background:**

Electronic health records (EHRs) have the potential to be used to produce detailed disease burden estimates. In this study we created disease estimates using national EHR for three high burden conditions, compared estimates between linked and unlinked datasets and produced stratified estimates by age, sex, ethnicity, socio-economic deprivation and geographical region.

**Methods:**

EHRs containing primary care (Clinical Practice Research Datalink), secondary care (Hospital Episode Statistics) and mortality records (Office for National Statistics) were used. We used existing disease phenotyping algorithms to identify cases of cancer (breast, lung, colorectal and prostate), type 1 and 2 diabetes, and lower back pain. We calculated age-standardised incidence of first cancer, point prevalence for diabetes, and primary care consultation prevalence for low back pain.

**Results:**

7.2 million people contributing 45.3 million person-years of active follow-up between 2000–2014 were included. CPRD-HES combined and CPRD-HES-ONS combined lung and bowel cancer incidence estimates by sex were similar to cancer registry estimates. Linked CPRD-HES estimates for combined Type 1 and Type 2 diabetes were consistently higher than those of CPRD alone, with the difference steadily increasing over time from 0.26% (2.99% for CPRD-HES vs. 2.73 for CPRD) in 2002 to 0.58% (6.17% vs. 5.59) in 2013. Low back pain prevalence was highest in the most deprived quintile and when compared to the least deprived quintile the difference in prevalence increased over time between 2000 and 2013, with the largest difference of 27% (558.70 per 10,000 people vs 438.20) in 2013.

**Conclusions:**

We use national EHRs to produce estimates of burden of disease to produce detailed estimates by deprivation, ethnicity and geographical region. National EHRs have the potential to improve disease burden estimates at a local and global level and may serve as more automated, timely and precise inputs for policy making and global burden of disease estimation.

## Introduction

Over 98% of the population in England are registered with a general practice. Almost all general practices use Electronic Health Records (EHRs) during their individual consultations to record clinical diagnoses, symptoms, lab results, tests, referrals to other specialties and prescriptions ^1^. In this analysis we also consider data captured during visits to secondary care hospitals, national disease registries and death records as EHRs.

These high coverage data could be used to generate automated, timely and detailed burden of disease estimates for national and local policy makers. The algorithms used to create such estimates could be made openly accessible allowing estimates to be widely, regularly and consistently produced. However, Global Burden of Disease studies in England found that accurate local data on mortality was better than for morbidity - a situation that is problematic for estimating burden for conditions with high levels of morbidity but low mortality such as low back and neck pain, skin and subcutaneous diseases, and depressive disorder ^2^. Primary care records linked to data from secondary care and mortality records may provide a comprehensive single source of consistently collected national data that can be used to give a detailed and consistent picture of disease burden over time for a diverse range of conditions.

National EHRs in England have been used extensively to conduct research studies ^3^ and these data are available for long time periods (for example, Hospital Episode Statistics from 1997, and Clinical Practice Research Datalink from 1987 ^1^). Whilst national primary care data coverage is high, there is a great deal of regional variation in the different clinical computer systems used ^4^. Additionally, the strengths and weaknesses of using linked national EHRs to produce stratified (e.g. by ethnicity, region or deprivation) disease burden estimates is uncertain.

In this study we aimed to address evidence gaps in estimating disease burden using national EHRs. To achieve this we had three objectives. First, to use a single source (primary care) and linked multi-source (primary care records linked to data from secondary care and mortality records) national EHRs to produce routine disease burden estimates for conditions with both high mortality (cancer) and high morbidity (diabetes and low back pain). Nearly everyone with these three conditions is registered with a GP and/or uses NHS acute services and as a result, will be included in Hospital Episode Statistics even if some treatment is provided in the private sector. Second, to compare these single source national EHR estimates of disease burden to existing incidence and prevalence data from other source estimates including disease registries and national cross-sectional health surveys. These comparisons enable us to triangulate our estimates with those currently used for public health and healthcare planning purposes. Our comparison estimates were selected based upon their strengths, including: high completion and coverage levels (e.g. National Cancer Registration and Analysis Service, Quality Outcomes Framework); high quality sampling strategies (e.g. Health Survey for England); high quality of the primary care data used as a result of multiple iterations of the data gathering process and training with participating general practices (e.g. Consultations in Primary Care Archive - CiPCA). Finally, to examine the ability of single source national EHR data to produce disease burden estimates over time and stratified by age, sex, ethnicity, deprivation and region.

## Methods

### Study design

We conducted a national population-based retrospective cohort study using EHRs to estimate the disease burden of four cancers (lung, colorectal, breast, prostate), diabetes (type I and II) and low back pain in England. Two cohorts were created; first a cohort containing data only from primary care records and second a nested cohort containing linked data from primary care, secondary care and national death certification registrations.

### Data resources

For the first cohort we used data from the Clinical Practice Research Datalink (CPRD GOLD), a large primary care database that contains anonymized electronic health records from more than 11 million people. We used records that were classified as acceptable for research having met previously specified data quality standards and shown to be broadly representative nationally ^1, 5^. Data within CPRD can relate to a consultation with a healthcare professional within primary care or describe other activities relating to the individual’s health or care including repeat prescriptions, lab test results or diagnoses made outside primary care. These data are recorded in the EHR using the Read code ^6^ system.

Through the CPRD linkage scheme, and where practices have provided consent, data were linked at person-level to inpatient data from Admitted Patient Care (APC) Hospital Episode Statistics (HES), a dataset containing details of all hospital admissions in England. Datasets were linked with a deterministic data linkage algorithm using date of birth, gender, postcode and NHS number (a unique ten digit numerical identifier assigned to NHS patients at birth or at first interaction with the healthcare system). Linkage was done by the Medicines and Healthcare products Regulatory Agency (MHRA) and NHS England with 88% of patients with research quality records having valid NHS number and eligible for linkage in the CPRD standard linked dataset release ^7^.

Data used to estimate cancer disease burden were also linked to mortality data from the Office for National Statistics (ONS) death certification register. Clinical diagnoses and procedures recorded during hospital admissions were recorded using the International Statistical Classification of Diseases and Related Health Problems, 10 ^th^ Revision (ICD-10) clinical classification system ^8^. We used the Index of Multiple Deprivation (IMD), linked by the MHRA, to be able to explore how disease burden estimates varied by relative deprivation in England ^9^.

### Inclusion/exclusion criteria

Our study population included male and female people registered at English general practices. Using these data, the study period we investigated was from 1 ^st^ January 2000 to 31 ^st^ December 2013. People contributed active follow up from the latest of: the date the primary care practice started to provide ‘Up-to-Standard’ data ^1^, the current registration date of the person, or the start of the study period. People stopped contributing active follow up from the earliest of: the last date CPRD collected data from the practice, the date the individual transferred out of the practice, date of death (recorded either in CPRD or ONS), or the end of the study. For prevalence estimates, the earliest date relating to a diagnostic read code found in either clinical, test and referral records or ICD code found within the active follow up period was used as the date of diagnosis.

***Case definitions for national EHRs.*** We expanded existing rule-based disease phenotyping algorithms to identify cases of cancer (breast, lung, colorectal and prostate), type 1 and 2 diabetes, and low back pain. Cancer diagnoses were determined using validated Read and ICD-10 terms as outlined by Bhaskaran *et al*. ^10^ Read and ICD-10 terms used to identify type 1 and 2 diabetes cases were based on work described by Eastwood *et al*. ^11^. Our low back pain estimates were derived using the same method and an expanded code list from Jordan ^12^ *et al*. 2014 with the addition of exacerbation of backache (16C8.00) and lumbalgia (N142.12) codes.

***Case definitions for comparison EHRs.*** Cancer incidence estimates were compared to those from the cancer registration statistics produced by the Office for National Statistics using the same method of standardisation (e.g. European Standard Population 2013 in 5-year age-band) to enable direct comparison ^13^. Table 1 summarises the sources of data used, case definitions, coverage and phenotypic depth of these cancer registry data. We used the following groups of ICD10 codes for our comparison estimates from the registry: C34 for lung, C18-C20 for bowel, C50 for breast cancer and C61 for prostate cancers. Our diabetes prevalence estimates were compared to those from estimates from Health Survey for England (HSE) and Quality Outcomes Framework (QOF). We applied HSE methods for estimating the annual diabetes prevalence. In survey year 2010 and for previous years of the survey, diabetes Type 2 was defined as being a self-reported diagnosis at aged 35 or older and not treated with insulin and Type 1 diagnosed before 35 and treated with insulin. For later surveys, 2011 and onwards, time of diagnosis was not used as a proxy measure to classify people by diabetes type. For QOF we used annual point-prevalence (percentage) for type 1 and 2 diabetes. Our low back pain estimates were compared to HSE and the Consultations in Primary Care Archive (CiPCA). The final algorithms used for all conditions are provided in the Extended Data File 2 ^14^.

**Table 1.** Existing data English and International sources for estimating population disease burden, their scale, strengths and weaknesses.

Data source	Description and coverage	Phenotypic depth	Case definition used in study	Scale	Strengths *	Weaknesses *
Hospital Episode Statistics (HES)	• Routinely collected hospital records • Available since 1987 • Emergency, inpatient and outpatient records	• Diagnosis using ICD10 coding • Age, gender, and ethnicity • Administrative information (i.e. waiting time, dates, and methods of admission and discharge)	See Appendices 2&3 for code lists used to create each case definition that are based on ICD10 codes building upon previously validated phenotypes.	• National - England	• National scale • Includes private practice information when carried out in NHS hospitals • Availability since 1987 • Possibility for linkage to primary care and vital statistics	• Primary collection rationale is financial • Accuracy dependent quality of coders which varies by location • Records relates to episodes not patients and may overestimate need if one patient has several episodes unless this is accounted for in analysis • Does not include private sector which accounts for high volumes of elective surgery in england
Clinical Practice Research Datalink	• Primary care electronic health records with linkage to secondary care settings, including disease registries and key demographic and socioeconomic datasets	• Diagnoses • Laboratory test results • Socio-demographic data • Prescriptions • Clinical referral information	Cancer (breast, lung, colorectal and prostate), type 1 and 2 diabetes, and low back pain. See Appendices 2&3 for code lists used to create each case definition that are based on Read and ICD10 codes building upon previously validated phenotypes.	• Data available from across the UK on approximately 6.9 % of the UK population since 1987	• Widely used for research on a national and international level with over 2,000 studies conducted to date • Linked data on hospital and death records available for subset • Long time series possible • Estimates can be produced for a wide range of conditions	• Coding based on Read system which is not used widely outside of UK and New Zealand and will be phased out in future • Prescription (i.e. the morbidity Read Code) is not directly linked to specific problems or events • Referral is not always directly linked to a specific problem • Under-recording of multiple problems presented at one contact
Health Survey for England	• Annual NHS-led survey collecting data on health, behaviour, social care, physical measures, mental health and wellbeing	• General health • Mental health • Smoking and alcohol consumption behaviour • Socioeconomic and demographic data • Height, weight and blood pressure, blood and saliva sample indicators • Use of health services	Diabetes Type 2 was defined as self-report of being diagnosed at aged 35 or older and not treated with insulin and Type 1 Diagnosed before 35 and treated with insulin. Chronic pain was defined in the HSE questionnaire as persistent pain or on and off pain in the last 3 months.	• Approximately 8,000 adults (16+) and 2,000 children (0–15) are interviewed each year.	• Availability since 1991 • Stratified random sample of the population • A response rate of around 60% is regularly achieved • Data have been linked for some years to HES	• Not linked to other EHRs • Based upon self report
National Disease Registration Service (NDRS)	• NDRS systematically collects data about cancer and tumour diseases in England from multiple data sources. NDRS includes data from histopathology and haematology services, HES, primary care including CPRD, radiotherapy, hospices, independent hospitals, screening services and death certificates.	• Patient personal data • Type of cancer • Stage of cancer • Treatment received • Histopathology and haematology records • Medical records (from GPs, national and independent hospitals) • Radiotherapy records • Hospice records • Screening service records • Death certificates • Data from other cancer registries	ICD10 codes were used for comparison estimates from the registry: C34 for lung, C18-C20 for bowel, C50 for breast cancer and C61 for prostate cancers.	• Approximately 300,000 cases annually	• Availability since 1990 • High completion and coverage	• England only • Time lag between collection and data availability
CiPCA	• Database of anonymised medical record data from a subset of general practices in North Staffordshire, UK, maintained by Keele University.	• Diagnoses • Laboratory test results • Socio-demographic data • Prescriptions • Clinical referral information	The case definition used Read codes that included codes with the terms ‘backache’ and ‘back pain’ with no region (lower or upper) stated, with the addition of exacerbation of backache and lumbalgia codes.	• Data from general practices that have a data-sharing agreement with the Research Institute.	• High quality data as a result of multiple iterations of the data gathering process and training recording of practices • Available since 2000	• Coding based on Read system which is not used widely outside of UK and New Zealand and will be phased out in future • Low volume of data • Prescription (i.e. the morbidity Read Code) is not directly linked to specific problems or events • Referral is not always directly linked to a specific problem • Under-recording of multiple problems presented at one contact
Quality Outcomes Framework (QOF)	• QOF is a quality management and analysis system for primary care • Primary purpose if for collection of data used to pay primary care practices against delivery for a set of milestones.	• Very limited as data only available in aggregate	Annual point-prevalence (percentage) for type 1 and 2 diabetes.	• UK wide and whilst voluntary coverage is high • Primary care practices reported in QOF will also be recording in CPRD	• Many conditions managed only in primary care and therefore QOF data can provide source of information for these conditions • Coverage is relatively complete as most people are registered with primary care • Data extracted monthly • Available since 2004	• Accuracy is dependent on coding of the disease registers • Data only available in aggregated form • Comparison across primary care practices not possible as different sizes and population characteristics affect the data.
Office for National Statistics (ONS) death certification register.	• Legal requirement for all deaths to be registered in 5 days	• Cause of death • Date and place • Dob, name, occupation • Details of spouse • Usual address	ICD Codes used for Cancer (C34 for lung, C18-C20 for bowel, C50 for breast cancer and C61 for prostate) and diabetes (E10, E11, E13, E14, N08, C10).	• National - UK with complete coverage due to legal status of reporting	• ICD 10 coding done by ONS, uniformly high quality • Complete and timely • Relatively accurate with uniform coding	• Clinical code less accurate for older patients with co- morbidities • ICD classification varies over years • Risk of bias in social class due to occupational advancement

*strengths and weaknesses specifically in relation to disease burden estimation

### Statistical analysis

We estimated the annual incidence of the four cancers studied, point-prevalence for diabetes and consultation period-prevalence for back pain for the period 2000–2013. For fair comparison with existing data sources and estimates, where possible we used methodology consistent with these existing data. Annual incidence and prevalence were estimated for all included people.

Population estimates were described for both cohorts and compared with the estimated population of England in 2013 ( Table 2). Analyses were stratified by age, sex, deprivation, ethnicity and region. We analysed data sub-regionally by using the nine statistical regions in England as defined by ONS. Quintiles of Index of Multiple Deprivation (IMD), at the person-level, were used as a measure of deprivation. Ethnicity was determined using methods previously described by Mathur *et al*. ^15, 16^ with further details provided in Extended Data File 2 ^14^. An available case analysis approach was used for all analyses. Analyses were performed using Stata version 14 SE and Stata version 15 SE. RWA, HE, AM, KB, RM, LS had access to the database population used for the study. 

**Table 2.** Demographic characteristics of CPRD people (January 2015 dataset build), and the subset of those active on 2nd July 2013, those with linked HES data, and those with IMD and ethnicity codes.

Characteristic	CPRD Active 2000–2013	CPRD-HES Active 2000–2013	CPRD Active 2013	CPRD-HES Active 2013	CPRD-HES IMD Active 2013	CPRD-HES Ethnicity Active 2013	CPRD-HES IMD & Ethnicity complete (Active 2013)	English national populations
Age								
N	7192977	5558552	3015525	2442065	2439473	1943896	1941925	53865817
<16	1463942 (20.4%)	1126066 (20.3%)	517793 (17.2%)	417611 (17.1%)	417115 (17.1%)	350337 (18.0%)	349914 (18.0%)	10209238 (19.0%)
16–24	805152 (11.2%)	630643 (11.3%)	342973 (11.4%)	277566 (11.4%)	277227 (11.4%)	195343 (10.0%)	195105 (10.0%)	6227694 (12.0%)
25–34	1215099 (16.9%)	937301 (16.9%)	343155 (11.4%)	279481 (11.4%)	279114 (11.4%)	227542 (11.7%)	227253 (11.7%)	7367357 (14.0%)
35–44	1096657 (15.2%)	844109 (15.2%)	466084 (15.5%)	376717 (15.4%)	376306 (15.4%)	300868 (15.5%)	300556 (15.5%)	7159067 (13.0%)
45–54	836456 (11.6%)	646864 (11.6%)	413696 (13.7%)	333833 (13.7%)	333523 (13.7%)	250788 (12.9%)	250585 (12.9%)	7543287 (14.0%)
55–64	696293 (9.7%)	539890 (9.7%)	398934 (13.2%)	323699 (13.3%)	323426 (13.3%)	248667 (12.8%)	248481 (12.8%)	6053995 (11.0%)
65–74	498357 (6.9%)	386360 (7.0%)	266094 (8.8%)	217082 (8.9%)	216905 (8.9%)	180454 (9.3%)	180324 (9.3%)	5023573 (9.3%)
75+	581021 (8.1%)	447319 (8.0%)	266796 (8.8%)	216076 (8.8%)	215857 (8.8%)	189897 (9.8%)	189707 (9.8%)	4281606 (7.9%)
Sex								
N	7192977	5558552	3015525	2442065	2439473	1943896	1941925	53865817
Male	3533680 (49.1%)	2726959 (49.1%)	1493530 (49.5%)	1206991 (49.4%)	1205721 (49.4%)	921460 (47.4%)	920544 (47.4%)	26533969 (49.0%)
Female	3659297 (50.9%)	2831593 (50.9%)	1521995 (50.5%)	1235074 (50.6%)	1233752 (50.6%)	1022436 (52.6%)	1021381 (52.6%)	27331848 (51.0%)
Region								
N	7192977	5558552	3015525	2442065	2439473	1943896	1941925	53865817
North East	141591 (2.0%)	109772 (2.0%)	62604 (2.1%)	50396 (2.1%)	50370 (2.1%)	42707 (2.2%)	42684 (2.2%)	2610481 (4.8%)
North West	937042 (13.0%)	752488 (13.5%)	450357 (14.9%)	370866 (15.2%)	369816 (15.2%)	305927 (15.7%)	305186 (15.7%)	7103260 (13.0%)
Yorkshire and the Humber	327539 (4.6%)	219753 (4.0%)	44891 (1.5%)	44710 (1.8%)	44698 (1.8%)	40160 (2.1%)	40149 (2.1%)	5337710 (9.9%)
East Midlands	341451 (4.7%)	192035 (3.5%)	31139 (1.0%)	23936 (1.0%)	23936 (1.0%)	16797 (.9.0%)	16797 (.9.0%)	4598729 (8.5%)
West Midlands	760046 (10.6%)	610672 (11.0%)	360797 (12.0%)	304894 (12.5%)	304604 (12.5%)	242889 (12.5%)	242668 (12.5%)	5674712 (11.0%)
East of England	870072 (12.1%)	675108 (12.1%)	276582 (9.2%)	237676 (9.7%)	237463 (9.7%)	174386 (9.0%)	174239 (9.0%)	5954169 (11.0%)
London	1189742 (16.5%)	919335 (16.5%)	523452 (17.4%)	422564 (17.3%)	422238 (17.3%)	368249 (18.9%)	367963 (18.9%)	8416535 (16.0%)
South East	1891845 (26.3%)	1413029 (25.4%)	932921 (30.9%)	682477 (27.9%)	682059 (28.0%)	508530 (26.2%)	508210 (26.2%)	8792626 (16.0%)
South West	733649 (10.2%)	666360 (12.0%)	332782 (11.0%)	304546 (12.5%)	304289 (12.5%)	244251 (12.6%)	244029 (12.6%)	5377595 (10.0%)
IMD								
N	5568975	5548979	2443152	2439473	2439473	1941925		
Q1	1226126 (22.0%)	1222640 (22.0%)	558189 (22.8%)	557590 (22.9%)	557590 (22.9%)	411172 (21.2%)		
Q2	1236889 (22.2%)	1233390 (22.2%)	552830 (22.6%)	552083 (22.6%)	552083 (22.6%)	436955 (22.5%)		
Q3	1113339 (20.0%)	1109963 (20.0%)	475235 (19.5%)	474548 (19.5%)	474548 (19.5%)	380938 (19.6%)		
Q4	1099285 (19.7%)	1093664 (19.7%)	474152 (19.4%)	472993 (19.4%)	472993 (19.4%)	387591 (20.0%)		
Q5	893336 (16.0%)	889322 (16.0%)	382746 (15.7%)	382259 (15.7%)	382259 (15.7%)	325269 (16.7%)		
Ethnicity								
N	4519253	4007643	2250736	1943896	1941925			53012456
White	3986336 (88.2%)	3551096 (88.6%)	1971141 (87.6%)	1703370 (87.6%)	1701608 (87.6%)			45281142 (85.0%)
South Asia	223818 (5.0%)	191111 (4.8%)	123357 (5.5%)	105599 (5.4%)	105551 (5.4%)			4143403 (7.8%)
Black	149170 (3.3%)	127322 (3.2%)	78275 (3.5%)	67652 (3.5%)	67555 (3.5%)			1846614 (3.5%)
Other	102745 (2.3%)	89514 (2.2%)	48128 (2.1%)	42125 (2.2%)	42085 (2.2%)			548418 (1.0%)
Mixed	57184 (1.3%)	48600 (1.2%)	29835 (1.3%)	25150 (1.3%)	25126 (1.3%)			1192879 (2.3%)

Table notes: Acceptable patients registered with English, CPRD, up-to-standard practices among male and females in our study period (2000 and 2013). *if active patients at mid-year are in their first year since current registration, these patients are excluded. Note: these are the patients that contribute to 1-year consultation prevalence and incidence estimates. There isn’t any publically available estimates for population by ethnicity for mid-2013 so 2011 was used. Data sources for national estimates: Age and Gender (mid-2013): https://www.ons.gov.uk/peoplepopulationandcommunity/birthsdeathsandmarriages/lifeexpectancies/adhocs/005676englishpopulationestimatesanddeathsbysexandsingleyearofage1993to2013 Region (mid-2013): via Population Estimates, Analysis Tool mid-2013: http://webarchive.nationalarchives.gov.uk/20160106144703/http://www.ons.gov.uk/ons/rel/pop-estimate/population-estimates-for-uk--england-and-wales--scotland-and-northern-ireland/2013/index.html Ethnicity (mid-2011 [census year]): was from the National Archives through (Nomis): https://www.nomisweb.co.uk/, then go to Data Downloads/Query Data/ Census 2011/Key Statistics All last accessed on 20th February 2018

***Cancer estimates: first incidence.*** Incidence was calculated by dividing the number of incident cases by the total active follow up time (in 100,000 person years) for lung, bowel, breast and prostate cancer. Follow up time ended at the time of the first cancer. Previous work has found evidence to suggest misclassification of prevalent cases, including cancer, as incident cases recorded in the first year after registration ^17^. For example, a healthcare professional registering a new person with their practice may enter the date of the primary care registration rather than the retrospective date of diagnosis, which leads to overestimation of incidence rates because the event occurred before registration and the corresponding years at risk were not included. In order to avoid this potential bias, active follow up was restricted to exclude the first year after registration for all incidence estimates ^18^. People identified with cancer before the active follow up period began were excluded from our analyses. We used direct age-standardization, standardizing to the European Standard Population 2013 in 5-year age-bands.

***Point-prevalence (estimates for diabetes).*** Annual point-prevalence of diabetes among those aged 17 years or older was calculated at mid-year (defined as July 2nd). The analysis was restricted to this age group to be consistent with QOF estimates. For a person to be included in the denominator for a given year they needed to be 17 years or older and be contributing active follow-up at mid-year. For a person to be included in the numerator they needed to be contributing to the active follow up and have at least one diagnosis Read code recorded before or at the mid-year point for that year ^11^. We calculated annual point prevalence by year of age for diabetes type 1 and 2 alone and for both types combined, the latter of which enabled comparisons with HSE and QOF estimates. When calculating prevalence estimates using linked CPRD-HES, we used CPRD dates and if diabetes was not recorded in CPRD but was recorded in HES, then the date of hospital admission recorded in HES was used.

***Period-prevalence (estimates for low back pain).*** Period prevalence, defined as a 1-year consultation prevalence, was estimated for lower back pain. The denominator was the total number of active people at mid-year (defined as 2nd July) for each year of the study. Among these people, where one or more consultation for back pain was recorded within an individual's active follow-up period, then the person would be counted once in the numerator. Consistent with the methodology applied to incidence, active follow-up was restricted to follow-up after one year from registration with the practice. People that were in their first year since primary care practice registration at mid-year were therefore excluded from the numerator and denominator for that year. Primary care records from CPRD were defined as consultation records if they were classified as either a face-to-face or telephone consultation (Extended Data File 2 ^14^) ^12, 19^. Primary care consultations were restricted to those that had a low back pain Read code in either their clinical, test or referral records. These additional codes did not exist at local level for this previous study. Secondary care admissions were restricted to those with an ICD-10 code ^12^ recorded with any diagnosis (code list given in Extended Data File 2 ^14^).

## Results

There were 7.2 million acceptable people contributing 45.3 million person-years of active follow-up between 2000–2013 ( Table 2; CPRD Active 2000–2013). Our nested primary care cohort (CPRD-HES Active 2000–2013) linked data to secondary care and death registry data and included 5.6 million people contributing 35.6 million person-years of follow-up between 2000–2013.

At mid-2013 there were approximately 3.0 million active CPRD individuals. This equates to 5.6% (3,015,525 / 53,865,817) of the English population. The median follow-up time for CPRD active people was 9.0 years (IQR 3.5 - 13.4). Of these active individuals, 2.4 million were linked to HES and of these linked people, 99.9% had Index of Multiple Deprivation (IMD) data recorded and 79.6% had ethnicity recorded.

In the following sections we describe our results for each condition in the following order. First, we present a description of our linked and unlinked estimates of disease burden for each disease over time. Second, we compare our new estimates of disease burden to existing incidence and prevalence data. Third, we describe the findings of stratified estimates (e.g. age, sex, region and IMD deprivation groups) from our results to comparator estimates.

### Cancer incidence

**Figure 1.**
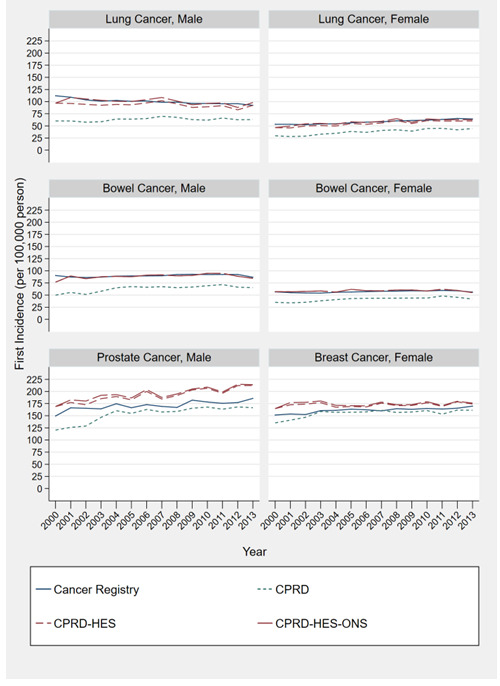
Directly standardized by age estimates of cancer for male and females (2000–2013) comparing estimates created using the following: 1) primary care records; 2) linked primary care and hospitalisation records; 3) linked primary care, hospitalisations and death records; and 4) National bespoke cancer registry records.

Estimates of incidence of lung, bowel, prostate and breast cancer using CPRD were consistently lower than CPRD-HES and CPRD-HES-ONS. The smallest difference of 12.4 per 100,000 people (170.4 vs 158.0) in breast cancer estimates in 2006, and the largest difference of 56.53 (182.6 vs 126.1) in prostate cancer in the year 2001 when comparing CPRD-HES-ONS to CPRD alone ( Figure 1).

CPRD-HES and CPRD-HES-ONS lung and bowel cancer estimates by sex were similar to cancer registry estimates. For prostate and breast cancer, CPRD-HES and CPRD-HES-ONS provided higher estimates than those produced by the cancer registry, with the greatest difference of 37.6 per 100,000 people (214.7 vs 177.1) found in prostate cancer estimates in 2012 (CPRD-HES-ONS).

**Figure 2.**
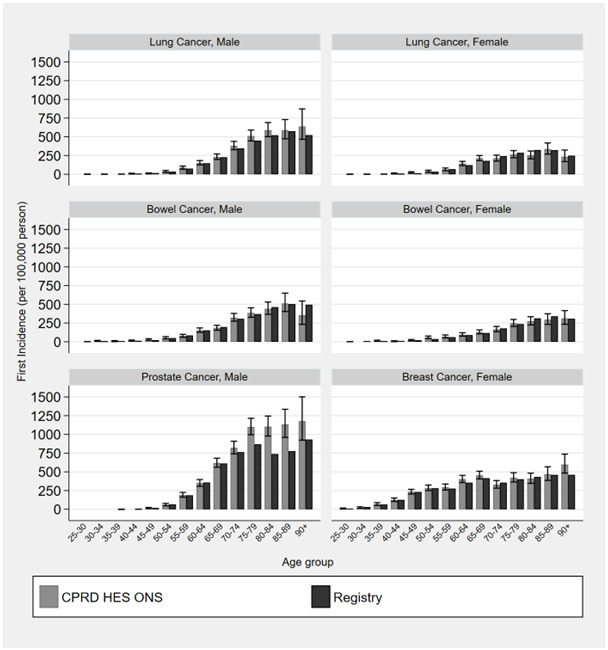
Incidence of cancer for male and females by age category comparing national bespoke cancer registry estimates (2013) to linked primary care, hospitalisations and death records. Error bars represent 95% confidence intervals.

Figure 2 provides cancer incidence using national EHR data compared to cancer registry estimates stratified by age. This shows that age-stratified estimates provide similar results to those created using registry data. The 95% confidence intervals of age categorised CPRD incidence estimates overlapped with registry estimates for women aged 35–39, lung cancer in women aged 40–44 and 45–49, breast cancer among women aged 45–50 and 60–64 and prostate cancer among men aged 80–84 (Extended data file 1 Figure S1 ^14^). All other unlinked CPRD incidence estimates by age were lower than the registry estimates (Extended data file 1 Figure S2 ^14^). We estimated the incidence of cancer by ethnicity, region and deprivation (IMD) for lung, colorectal, breast and prostate (Extended data file 1 Figures S3 – S6 ^14^).

### Diabetes

**Figure 3.**
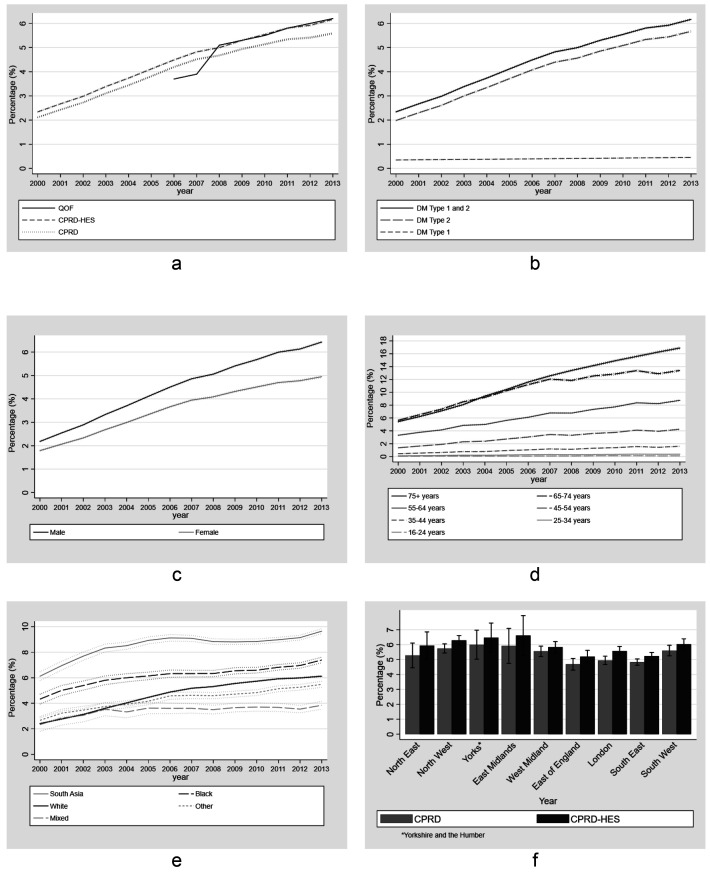
Annual point-prevalence for diabetes comparing national primary care quality outcome framework data to linked CPRD-HES for ( **a**) Type 1 and Type 2 combined ( **b**) Type 1 and Type 2 combined and separate; ( **c**) Type 2 by gender, ( **d**) Type 2 by age group, ( **e**) Type 2 by ethnicity (Error bars represent 95% confidence intervals) and ( **f**) Type 2 by region comparing unlinked CPRD and linked CPRD-HES (Error bars represent 95% confidence intervals).

Point-prevalence estimates using CPRD and CPRD-HES for combined type 1 and type 2 diabetes increased steadily over time. For CPRD data, this increase was from 2.1% (95%CIs: 2.0 - 2.2%) in 2000 to 5.6% (95%CIs: 5.5 - 5.7%) in 2013 ( Figure 3a). Linked CPRD-HES estimates for combined type 1 and type 2 diabetes were consistently higher than those of CPRD alone, with the difference steadily increasing over time from 0.26% (2.99% for CPRD-HES vs. 2.73% for CPRD) in 2002 to 0.58% (6.17% vs. 5.59%) in 2013. Point prevalence estimates of combined type 1 and type 2 diabetes using CPRD-HES increased from 2.3% (95%CI 2.2 - 2.5%) in 2000 to 6.2% (95%CI 6.0 - 6.3%) in 2013. Point prevalence estimates for diabetes type 2 using CPRD-HES increased steadily from 2.0% (95%CI 1.8 - 2.1%) in 2000 to 5.7% (95% CI 5.5 - 5.8%) in 2013.

In 2006 and 2007, QOF estimates were considerably lower than CPRD and CPRD-HES estimates (the largest difference in 2007 of 0.62% and 0.92%, respectively), but from 2008 to the end of the study period QOF estimates were similar to CPRD-HES estimates. We compared our estimates to those from the Health Survey for England (HSE) study to CPRD and CPRD-HES. HSE showed an increase in type 2 prevalence but did not show a similar increase in type 1 (Extended Data File 2 ^14^). HSE weighted estimates for type 1 and type 2 diabetes were very similar over time to our CPRD-HES estimates. For type 1 diabetes, CPRD-HES estimates were lower than HSE by 0.1% (0.4% vs 0.5%) in 2010 and 2011, and higher by 0.1% (0.5% vs 0.4%) in 2012. For type 2 diabetes, CPRD-HES estimates were lower than HSE in 2010 and 2011 (by 0.1% - 5.1% vs 5.2%- and 0.2% - 5.3% vs 5.5% - respectively) and the same as HSE in 2012 (5.4%) and 2013 (5.7%).

Between 2000 and 2013, prevalence of diabetes type 2 increased over time for each age group ( Figure 3d). Prevalence was similar between the 65–74 and 75+ age groups up to 2007 (0.5% greater among 75+ in 2007 - 12.5% vs 12.0%). After 2007 there was a reduction in the relative increase of prevalence over time and by 2013 the difference between these age groups was greatest and 3.5% greater in the 75+ age group (16.9% vs 13.4%). Prevalence of type 2 was highest in the most deprived quintile for IMD and increased more in this group compared to the least deprived, widening the difference between these quintiles over time. Prevalence in the least deprived group increased from 1.5% (95%CI, 1.2 - 1.8%) in 2000 to 4.8% (95%CI 4.3 - 4.8%) in 2013 and from 2.5% (95%CI 2.1 - 2.8%) to 6.9% (95%CI 6.5 - 7.2%) in the most deprived group. Prevalence of Diabetes Type 2 increased in all ethnic groups over time. South Asians had the highest prevalence across all years. The groups with the highest increase during the study period were White, South Asian and Black with a 3.7%, 3.5% and 3.1% increase respectively between 2000 and 2013. Geographically, prevalence was generally higher in the North of England and the Midlands compared to the South and East of England. Prevalence estimates of type 2 diabetes using CPRD and CPRD-HES showed similar patterns by region in 2013 ( Figure 3f).

### Low back pain

**Figure 4.**
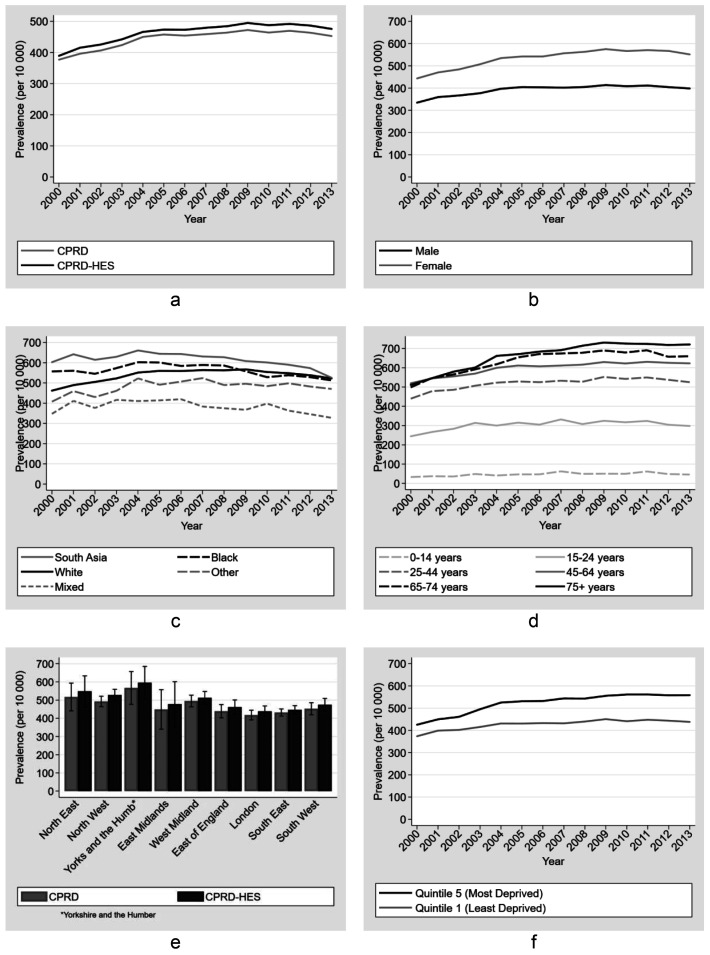
Prevalence estimates (per 10 000 person) for Low Back Pain (LBP) by ( **a**) source, ( **b**) gender, ( **c**) ethnicity, ( **d**) age group, ( **e**) region and ( **f**) Index of Multiple Deprivation (IMD). Primary care records used for all figures except ( **a**) and ( **e**) where unlinked primary care and linked primary care and hospitalisation records were used (Error bars represent 95% confidence intervals.).

We observed a small increase in low back pain consultation prevalence when comparing CPRD-HES to CPRD estimates alone ( Figure 4a). In 2013 the prevalence was 476 (95%CI 464 - 488) for CPRD-HES and 453 (95%CI 442 -464) per 10 000 persons using CPRD alone. Low back pain prevalence increased over time but appeared to plateau in 2008 and was followed by a small decrease from 495 (95%CI 483 - 506) per 10 000 persons in 2009 to 476 (95%CI 464 - 488) in 2013 (CPRD-HES).

**Figure 5.**
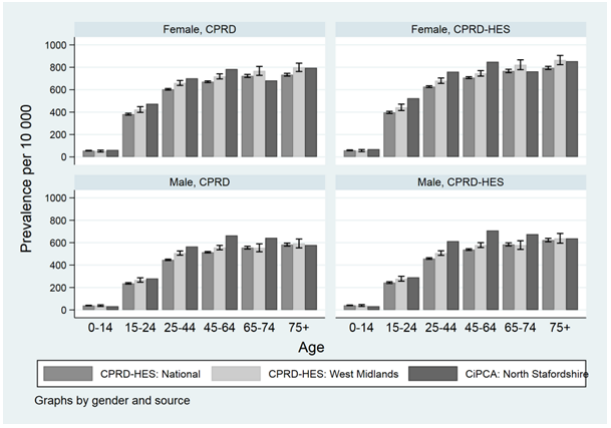
1-year consultation prevalence for low back pain nationally, the West Midlands and Consultations in Primary Care Archive for North Staffordshire (CiPCA; includes regional data from North Staffordshire general practices) in 2010 using primary care records with secondary care data in CIPCA recorded by primary care from hospital correspondance. Error bars represent 95% confidence intervals. Note: CiPCA Estimates are from Jordan *et al*. 2014 ^12^.

Prevalence of low back pain increased with age using national CPRD and CPRD regional data for the West Midlands ( Figure 5). Our prevalence estimate of low back pain for the CPRD region of West Midlands were generally consistent with 95% confidence intervals overlapping estimates from CiPCA. Low back pain estimates increased up to age group 45-64 for males and females for our national EHR (CPRD-HES) estimates and CiPCA.

Prevalence estimated using linked CPRD-HES data was 38% greater among women (prevalence ratio 1.38 (95%CI 1.37 - 1.40) with a prevalence difference of 153.6 per 10,000 people (534.3 vs 380.8) compared to men in 2013 - a trend that was consistent over time. Between 2000 and 2013 there was an overall increase in prevalence among those with white and ‘other' ethnicity, while there was a decline among those with south asian, black and mixed ethnicity, with black, south asian and white ethnicities becoming similar in 2013 ( Figure 4c). The largest difference between any two ethnic groups was 252.5 per 10,000 people, or 67% (PR 1.67 (95%CI 1.54 - 1.82)), between the south asian and mixed ethnicities in 2008 (627.4 vs 374.9). This difference was consistent across time. Prevalence in the most deprived quintile increased over time between 2000 and 2013 ( Figure 4f), with the largest difference of 27% (PR 1.27 (95%CI 1.25 - 1.30; 558.7 vs 438.2) in 2013 compared to the least deprived quintile. In 2013, prevalence was higher in the North of England compared to the South regions of England, but confidence intervals were overlapping except for comparisons between the North West and Yorkshire and Humber compared to London. For these categories, the respective CPRD-HES prevalence differences with London were 89.6 per 10,000 people and 156.8, or 20% (PR 1.20 (95%CI 1.18 - 1.23); 527.9 vs 438.3; North West) and 36% (PR 1.36 (95%CI 1.31 - 1.41); 595.2 vs 438.3; Yorkshire and Humber) greater than London ( Figure 4e).

## Discussion

Our study uses national EHRs for routine surveillance of disease burden. We used data from more than seven million people to estimate disease burden for three diverse conditions that have either high levels of morbidity, mortality or both. Our results address important evidence gaps in estimating disease burden estimation using a single source of national electronic health records and meet our three study objectives.

First, we have used national EHRs to produce estimates for conditions with a high mortality such as cancer and high morbidity such as diabetes and low back pain. We undertook this work building upon existing validated disease phenotypes and applied these to national EHRs. In future it will be possible to relatively quickly reapply our code to update estimates of these three sets of conditions, but also expand on the number of phenotypes to provide more comprehensive estimates for a wider range of conditions. With the move to SNOMED ^20^ coding lists in primary care - a structured clinical vocabulary for use in an electronic health record - Read code morbidity lists will need to be converted to SNOMED.

Second, for cancer and diabetes, we have compared our single source national EHR estimates of disease burden to current national incidence and prevalence data that have high completion and coverage (e.g. National Cancer Registration and Analysis Service, Quality Outcomes Framework) and shown that these estimates are broadly comparable. Our prostate and breast cancer were higher than cancer registry estimates, which may reflect the true burden of disease as a result of under-reporting of these common conditions in the cancer register, or they may be false over-estimates. Falsely inflated results may occur due to misclassification bias within primary care records. For example, positive prostate specific antigen results may initially be incorrectly classified as prostate cancer in primary care records, but actually be due to benign prostate enlargement. This misclassification is less likely to occur within the cancer registry data. Read codes used to record diagnoses in primary care can be recorded using non-specific codes; e.g. codes that specify back pain rather than low or upper back pain or diabetes rather than type 1 and type 2 diabetes. In order to estimate specific and more meaningful morbidity prevalence estimates, we encourage GPs to record completely and accurately. Our CPRD-HES estimates for type 1 and type 2 diabetes were very similar over time to those from Health Survey for England which is produced using self-reported data from a stratified random sample of the population. Our prevalence estimates for lower back pain were generally consistent with estimates from CiPCA, which includes high quality primary care data as a result of multiple iterations of the data gathering process and training with participating general practices. Our results are also consistent with more recently published data ^21^.

Finally, we have demonstrated that national EHR data from across healthcare settings can produce disease burden estimates over time and stratified by age, sex, ethnicity, deprivation and region. Our results provide national estimates of lower back pain, and in addition to this, provide important data on subgroups. Our data indicate that South Asian and Black ethnicities had highest prevalence estimates for low back pain and that the burden was highest in the lower socio-economic groups.

A weakness of the data used are that they are derived from one primary care computer system, which is distributed unevenly across the UK with low representation in the north-east and midlands. Future work should develop a framework (a governance framework and a harmonised data model) to enable these studies to be run on general practice databases from all major systems. We did not cluster by general practice in the construction of the confidence intervals, which would have resulted in wider confidence intervals than those currently presented. There were high levels of missing data for ethnicity, and our data were overrepresented for white, and underrepresented for south asians and black populations. Our results showed variation by ethnicity and as a result our national and age group estimates, which did not control for ethnicity, may be biased towards levels found in white ethnic groups. Missing data appeared not to have caused substantial bias in the comparison of EHR-based estimates versus those from national registries or survey in the outcomes being investigated in this work. However, missing data could be an issue with other EHRs, even in high population coverage settings, when certain variables are not missing completely at random, which should be considered when exploring the potential automated derivation of disease burden estimates from EHRs. Secondary, tertiary, or further illness or health conditions in patients with high levels of co-morbidities may not be comprehensively recorded in busy general practice consultations, especially for elderly patients or patients with difficulties with communicating. EHR-based prevalence or incidence estimates of these illnesses or conditions, and for certain subgroups of patients, may be underestimated in the EHR as a result.

Our proposed methods have several strengths over existing disease burden data sources. Compared to cross-sectional surveys, our methods do not rely on self-reported outcomes, are clinician coded, and once validated, could be produced at lower annual costs on a routine basis. The data are national in scale and are detailed enough to produce small local area estimates without the need for substantial statistical modelling.

A recently conducted systematic review examined the challenges and solutions of using electronic health records as based disease surveillance systems ^22^. The review found that many challenges exist including the amount of time and investment required to set up such systems and the privacy and security challenges of such approaches. However, it also identified potential opportunities including the use of machine learning algorithms for unstructured data and appropriate technical solutions for data processing. Other studies have compared the results of electronic health record disease surveillance systems to survey results and found that they can produce robust estimates of smoking and obesity prevalence in the general population ^23^. Other UK and international studies have examined the use of electronic disease surveillance for specific diseases including musculoskeletal conditions ^24^, chronic disease ^25^ and cardiovascular disease ^26^ and described their use in these specific settings. 

In the UK, a growing number of studies are using primary care data linked to HES data. It is encouraging that the data are broadly representative nationally and that estimates from this study provide temporal trends comparable with other work to-date. Our data support a growing body of evidence from other studies for the validity of national EHRs for estimating disease burden from a diverse range of conditions including cardiovascular disease ^27, 28^, bronchiectasis ^29^, obesity, and learning disabilities ^30^ and a chronological map of 308 physical and mental health conditions ^31^. This growing body of evidence suggests that this approach is generalisable to a wide range of conditions and we envisage that health data providers such as CPRD and NHS England will increasingly be able to provide data within shorter periods of time for pre-approved studies. Expanding this approach could enable continuous collection of registry data for some conditions, instead of using intermittent validation surveys, or augmenting national EHR where needed with individual or clinician reported information. Linked EHRs could be used to produce a picture of the individual journey through primary care and secondary care and these results would allow opportunities for primary, secondary and tertiary prevention to be identified and acted upon. Population level estimates of multiple morbidity, for which we currently lack data in England, could also be produced using these data. In countries with health systems with high population coverage and complete linked electronic health records, automated, timely and precise disease burden estimates could potentially be available based on use of existing data.

## Data Availability

This study used data provided by the CPRD for health research purposes. Access to CPRD data, including UK Primary Care Data, and linked data such as Hospital Episode Statistics, is subject to protocol approval via CPRD’s Research Data Governance (RDG) Process. Estimating disease burden using national linked electronic health records: a study using an English population-based cohort. https://doi.org/10.5281/zenodo.7895913 ^14^. This project contains the following extended data: Extended Data File 1: Supplementary Figures Extended Data File 2: Supplementary Tables Data are available under the terms of the Creative Commons Zero "No rights reserved" data waiver (CC0 1.0 Public domain dedication).
