# Supplementary material for: Estimating disease burden using national linked electronic health records: a study using an English population-based cohort
Source: Wellcome Open Res. 2024 Jun 19;8:262. Originally published 2023 Jun 21. [Version 2] doi: 10.12688/wellcomeopenres.19470.2 (PMC11292189; doi:10.12688/wellcomeopenres.19470.2)
Supplement: Peer review history

# Reviewer report: Reviewer response for version 2

**DOI:** 10.21956/wellcomeopenres.23730.r87196

**Published:** 2024-07-31

**Authors:** Joyce C Ho

**Affiliations:** 1 Emory University, Atlanta, USA

Thanks for adding the paragraph describing global disease surveillance efforts. I have no further comments to make.

Is the work clearly and accurately presented and does it cite the current literature?

Partly

If applicable, is the statistical analysis and its interpretation appropriate?

Yes

Are all the source data underlying the results available to ensure full reproducibility?

No

Is the study design appropriate and is the work technically sound?

Yes

Are the conclusions drawn adequately supported by the results?

Yes

Are sufficient details of methods and analysis provided to allow replication by others?

Yes

Reviewer Expertise:

Machine learning, Electronic health records

I confirm that I have read this submission and believe that I have an appropriate level of expertise to confirm that it is of an acceptable scientific standard.

# Reviewer report: Reviewer response for version 2

**DOI:** 10.21956/wellcomeopenres.23730.r87195

**Published:** 2024-06-20

**Authors:** Thaddäus Tönnies

**Affiliations:** 1 Leibniz Center for Diabetes Research at the Heinrich Heine University, Düsseldorf, Germany

Thanks very much for your detailed response. I have no further comments.

Is the work clearly and accurately presented and does it cite the current literature?

Yes

If applicable, is the statistical analysis and its interpretation appropriate?

Yes

Are all the source data underlying the results available to ensure full reproducibility?

No

Is the study design appropriate and is the work technically sound?

Yes

Are the conclusions drawn adequately supported by the results?

Yes

Are sufficient details of methods and analysis provided to allow replication by others?

Yes

Reviewer Expertise:

Epidemiology

I confirm that I have read this submission and believe that I have an appropriate level of expertise to confirm that it is of an acceptable scientific standard.

# Reviewer report: Reviewer response for version 1

**DOI:** 10.21956/wellcomeopenres.21567.r67551

**Published:** 2023-10-19

**Authors:** Joyce C Ho

**Affiliations:** 1 Emory University, Atlanta, USA

The article assesses the use of electronic health records (EHRs) to produce disease burden estimates. Using existing disease phenotypes for 4 types of cancer, type 1 and type 2 diabetes, and lower back pain, the EHR-based estimates are compared to disease registries and national cross-section health surveys. The article also examines the disease burdens stratified by age, sex, ethnicity, deprivation, and region. The results suggest that the estimates are consistent with existing collection mechanisms and can offer more timely and precise inputs for estimating disease burden.

 The article overall is well-written and the statistical analysis and methods are appropriate, especially the use of existing, validated disease phenotypes to construct the disease estimates from EHR. Unfortunately, access to the primary care EHR data is not available due to privacy concerns, so the study is not fully reproducible but sufficiently described that the process could replicated.

 One limitation of the article is the lack of discussion compared to other EHR-based disease surveillance efforts worldwide. Two notable omissions include a recent survey on EHR-based surveys and estimating musculoskeletal conditions (the latter one seemingly uses the same dataset). There are also 3 works linked below while not necessary would also be interesting to compare/contrast the article findings against. Since these citations focus on the United States, not all findings will be relevant/repeatable given differences in EHR data collection, but discussions of EHR data quality and other insights might be portable to better understand how generalizable it is to other countries.

Is the work clearly and accurately presented and does it cite the current literature?

Partly

If applicable, is the statistical analysis and its interpretation appropriate?

Yes

Are all the source data underlying the results available to ensure full reproducibility?

No

Is the study design appropriate and is the work technically sound?

Yes

Are the conclusions drawn adequately supported by the results?

Yes

Are sufficient details of methods and analysis provided to allow replication by others?

Yes

Reviewer Expertise:

Machine learning, Electronic health records

I confirm that I have read this submission and believe that I have an appropriate level of expertise to confirm that it is of an acceptable scientific standard, however I have significant reservations, as outlined above.

## References

[1] : Electronic health record-based disease surveillance systems: A systematic literature review on challenges and solutions. *J Am Med Inform Assoc* .2020;27(12) : 10.1093/jamia/ocaa186 1977-1986 10.1093/jamia/ocaa186 32929458 PMC7727341

[2] : Estimating the population health burden of musculoskeletal conditions using primary care electronic health records. *Rheumatology (Oxford)* .2021;60(10) : 10.1093/rheumatology/keab109 4832-4843 10.1093/rheumatology/keab109 33560340 PMC8487274

[3] : New Directions in Public Health Surveillance: Using Electronic Health Records to Monitor Chronic Disease. *J Public Health Manag Pract* .28(2) : 10.1097/PHH.0000000000001501 203-206 10.1097/PHH.0000000000001501 35100219

[4] : Establishing a National Cardiovascular Disease Surveillance System in the United States Using Electronic Health Record Data: Key Strengths and Limitations. *J Am Heart Assoc* .2022;11(8) : 10.1161/JAHA.121.024409 e024409 10.1161/JAHA.121.024409 35411783 PMC9238467

[5] : Can Electronic Health Records Be Used for Population Health Surveillance? Validating Population Health Metrics Against Established Survey Data. *EGEMS (Wash DC)* .2016;4(1) : 10.13063/2327-9214.1267 1267 10.13063/2327-9214.1267 28154837 PMC5226379

# Response

**Published:** 2024-04-05

**Authors:** Robert Aldridge

**Affiliations:** UCL, UK

Review comment: “One limitation of the article is the lack of discussion compared to other EHR-based disease surveillance efforts worldwide. Two notable omissions include a recent survey on EHR-based surveys and estimating musculoskeletal conditions (the latter one seemingly uses the same dataset). There are also 3 works linked below while not necessary would also be interesting to compare/contrast the article findings against. Since these citations focus on the United States, not all findings will be relevant/repeatable given differences in EHR data collection, but discussions of EHR data quality and other insights might be portable to better understand how generalizable it is to other countries.”

 Author response: In response to this helpful comment, we have added a paragraph of further discussion on this topic describing global analyses that have examined disease surveillance efforts, including the five helpful references provided by the reviewer.

# Reviewer report: Reviewer response for version 1

**DOI:** 10.21956/wellcomeopenres.21567.r62082

**Published:** 2023-08-09

**Authors:** Thaddäus Tönnies

**Affiliations:** 1 Leibniz Center for Diabetes Research at the Heinrich Heine University, Düsseldorf, Germany

Aldridge et al. estimated the burden of several diseases in terms of incidence and prevalence using electronic health records linked to additional data sources. The long-term goal is to provide a data source of consistently collected national data that can be used to give a consistent, automated, and timely picture of disease burden over time for a diverse range of conditions. Overall, the paper is clearly written and presents interesting results which are relevant for decision makers and the scientific community working with EHR. I only have some minor comments.

1. According to the authors, one advantage of EHR is the potential to provide timely results. However, the data in this study are rather dated (2000 to 2013). I think this is totally fine for this study, as it appears more concerned with feasibility than with substantive results. Nevertheless, it would be interesting to have some information on how timely results might be available in future, one the procedures in this study are routinely implemented.
2. Is there a rationale for estimating incidence for cancer, but prevalence for diabetes and low back pain? Results for the incidence of diabetes and low back pain would also very relevant. In fact, in would be a strength of this study if incidence of diabetes could also be estimated, since data on prevalence is more often available.
3. During linkage, the sample size substantially decreased from 11 million to 2.4 million. Please specify what led to this reduction, whether this could introduce selection bias and if representativeness can still be assumed. Also discuss whether the difference between linked and unlinked estimates of disease burden could be due to this selection rather than improved detection of cases.
4. The authors stratified the data to obtain stratum-specific estimates. A probably more efficient approach could be to use regression models with the stratifying variables as (continuous) predictors. This might also overcome problems with small sample sizes in some strata (only strata with at least 5 cases were analyzed).
5. The results were compared to published estimates. Please mention if one of the comparators can be considered the “gold standard” or most valid source.
6. Figure 1, 3, 5: I suggest to add confidence intervals to the lines.
7. Figure 2, 4: Please specify in the legend, what the error bars represent.
8. Figure 4: Please specify in the legend, to which disease the prevalence refers.

Is the work clearly and accurately presented and does it cite the current literature?

Yes

If applicable, is the statistical analysis and its interpretation appropriate?

Yes

Are all the source data underlying the results available to ensure full reproducibility?

No

Is the study design appropriate and is the work technically sound?

Yes

Are the conclusions drawn adequately supported by the results?

Yes

Are sufficient details of methods and analysis provided to allow replication by others?

Yes

Reviewer Expertise:

Epidemiology

I confirm that I have read this submission and believe that I have an appropriate level of expertise to confirm that it is of an acceptable scientific standard, however I have significant reservations, as outlined above.

# Response

**Published:** 2024-04-05

**Authors:** Robert Aldridge

**Affiliations:** UCL, UK

Thank you very much for your helpful comments which we respond to below:

 Review comment: “According to the authors, one advantage of EHR is the potential to provide timely results. However, the data in this study are rather dated (2000 to 2013). I think this is totally fine for this study, as it appears more concerned with feasibility than with substantive results. Nevertheless, it would be interesting to have some information on how timely results might be available in future, one the procedures in this study are routinely implemented.”

 Author response: The reviewer correctly identifies that the study is a feasibility study hence it does not impact our results. We agree that it would be useful to have more timely results in future. It is possible that the data could be provided every month, quarter or year from the data provider – CPRD – if permissions were sought and approved, which would provide a useful close to real-time monitoring mechanism.

 Review comment: “Is there a rationale for estimating incidence for cancer, but prevalence for diabetes and low back pain? Results for the incidence of diabetes and low back pain would also very relevant. In fact, in would be a strength of this study if incidence of diabetes could also be estimated, since data on prevalence is more often available.”

 Author response: We estimated incidence for cancer, but prevalence for diabetes and low back pain to make our results comparable with previous published estimates. In this paper, for cancer we compare with the national cancer registry. For diabetes, we compare with the National Health Survey for England. For back pain, we use consultation prevalence (percentage of patient that have had at least one consultation about backpain in a year), similar to Jordan (reference 12). This is because historically there is a lag between having back pain and seeing the GP and therefore it is difficult to know both start and end date of back pain using GP and hospital records. We agree with the reviewer that incidence for diabetes could be calculated using these data, along with prevalence of cancer. We agree that these estimates would be useful in future studies.

 Review comment: “During linkage, the sample size substantially decreased from 11 million to 2.4 million. Please specify what led to this reduction, whether this could introduce selection bias and if representativeness can still be assumed. Also discuss whether the difference between linked and unlinked estimates of disease burden could be due to this selection rather than improved detection of cases."

 Author response: Table 2 describes, the demographic distribution of patients registered with practices contributing active follow-up to CPRD and the subset with linked data. Table 2 shows that the subset of participants with linked data have broadly similar demographic distributions to those without linked data. The differences in our estimates of disease burden between linked and unlinked data are more likely driven by the differences in health care utilization and clinical practice for the conditions in our study (e.g. cancer is much more commonly managed in hospital not primary care and therefore cases are more likely to appear in these hospital records) rather than any smaller selection bias introduced through linkage.

 Review comment: “The authors stratified the data to obtain stratum-specific estimates. A probably more efficient approach could be to use regression models with the stratifying variables as (continuous) predictors. This might also overcome problems with small sample sizes in some strata (only strata with at least 5 cases were analyzed).

 Author response: We have removed the sentence “Analyses by strata were restricted to those with 5 incident/prevalent cases or more” as we had sufficient sample size that this was not an issue in our final analysis. As a result, we also feel that our approach is appropriate. Future analyses could consider using regression models with the stratifying variables as (continuous) predictors, but unfortunately, we would not be able to do this for our current analysis as we are no longer able to access underlying data.

 Review comment: “The results were compared to published estimates. Please mention if one of the comparators can be considered the “gold standard” or most valid source. .”

 Author response: We deliberately avoided the use of the word “gold standard” in our manuscript as we believe no such “gold standard” data source exists for this setting and study. Instead, our estimates were compared to the highest quality and most appropriate existing data.

 Review comment: “Figure 1, 3, 5: I suggest to add confidence intervals to the lines. .”

 Author response: Figures 3 and 5, included 95% confidence intervals where possible, but these were not labelled correctly. Thank you for noting this omission – we have updated the figure title to accurately reflect this point.

 Review comment: “Figure 2, 4: Please specify in the legend, what the error bars represent.”

 Author response: Thank you for noting this omission - Error bars represent 95% confidence intervals which we have now updated in the figure title.

 Review comment: “Figure 4: Please specify in the legend, to which disease the prevalence refers.”

 Author response: Thank you for spotting this omission. We have clarified that the prevalence refers to low back pain.
